# Perioperative lung protective ventilation in obese patients

**DOI:** 10.1186/s12871-015-0032-x

**Published:** 2015-05-06

**Authors:** Ana Fernandez-Bustamante, Soshi Hashimoto, Ary Serpa Neto, Pierre Moine, Marcos F Vidal Melo, John E Repine

**Affiliations:** 1 Department of Anesthesiology, University of Colorado SOM, Aurora, CO USA; 2 Department of Anesthesiology and Webb-Waring Center, University of Colorado SOM, Aurora, CO USA; 3 Department of Anesthesia, Critical Care and Pain Medicine, Massachusetts General Hospital, Boston, MA USA; 4 Department of Critical Care Medicine, Hospital Israelita Albert Einstein, São Paulo, Brazil; 5 Department of Intensive Care, Academic Medical Center, University of Amsterdam, Amsterdam, The Netherlands; 6 Department of Medicine, University of Colorado SOM, Aurora, CO USA

**Keywords:** Lung protective ventilation, Obese surgical patients, Mechanical ventilation, Perioperative ventilation, Obesity

## Abstract

The perioperative use and relevance of protective ventilation in surgical patients is being increasingly recognized. Obesity poses particular challenges to adequate mechanical ventilation in addition to surgical constraints, primarily by restricted lung mechanics due to excessive adiposity, frequent respiratory comorbidities (i.e. sleep apnea, asthma), and concerns of postoperative respiratory depression and other pulmonary complications. The number of surgical patients with obesity is increasing, and facing these challenges is common in the operating rooms and critical care units worldwide. In this review we summarize the existing literature which supports the following recommendations for the perioperative ventilation in obese patients: (1) the use of protective ventilation with low tidal volumes (approximately 8 mL/kg, calculated based on predicted -not actual- body weight) to avoid volutrauma; (2) a focus on lung recruitment by utilizing PEEP (8–15 cmH_2_O) in addition to recruitment maneuvers during the intraoperative period, as well as incentivized deep breathing and noninvasive ventilation early in the postoperative period, to avoid atelectasis, hypoxemia and atelectrauma; and (3) a judicious oxygen use (ideally less than 0.8) to avoid hypoxemia but also possible reabsorption atelectasis. Obesity poses an additional challenge for achieving adequate protective ventilation during one-lung ventilation, but different lung isolation techniques have been adequately performed in obese patients by experienced providers. Postoperative efforts should be directed to avoid hypoventilation, atelectasis and hypoxemia. Further studies are needed to better define optimum protective ventilation strategies and analyze their impact on the perioperative outcomes of surgical patients with obesity.

## Introduction

Proper ventilatory settings have a proven impact on clinical outcomes in Intensive Care Unit (ICU) patients with or without risk for the Acute Respiratory Distress Syndrome (ARDS) [1,2]. While lung protective ventilation with low tidal volumes (V_T_) and the use of positive end-expiratory pressure (PEEP) are now considered routine for ICU patients, the implementation of protective ventilation strategies in the operating room is not widespread [3-5]. These practices may reflect the shortage of convincing prospective trials showing a significant negative impact of non-protective ventilation of short duration on clinical outcomes of patients with healthy lungs. However, the relevance of optimal mechanical ventilation for surgical patients during general anesthesia is being increasingly recognized. Recent studies [6-8] and meta-analyses [9,10] suggest that intraoperative ventilatory practices may contribute not only to ARDS but also to the development of other postoperative pulmonary complications. Although postoperative ARDS is rare in patients at low risk, postoperative pulmonary complications including atelectasis, pneumonia, or respiratory failure, can occur in up to 40% in high-risk patients [11] and are associated to worse surgical outcomes [12].

Adequate ventilation of the surgical patient with obesity is particularly challenging because of the unique loads to lung mechanics posed by excessive restrictive adiposity, the common presence of additional respiratory morbidities such as asthma or sleep apnea, and/or concerns regarding postoperative respiratory depression related to the altered pharmacokinetics with increased adiposity. The real-to-predicted body weight disparity in obese patients and the unique use of height, instead of weight, in formulas used for tidal volume calculation based on predicted body weight [1] undoubtedly contribute to obesity being a recurrent risk factor for receiving inappropriately large tidal volumes during mechanical ventilation [3,5,13].

Ventilating obese patients is becoming a frequent challenge since the prevalence of obesity is steadily increasing and reaching epidemic proportions worldwide [14,15]. Fitucane *et al.* [15] found a worldwide average increase in age-standardized Body Mass Index (BMI, defined as weight (kg)/height (m)^2^) of 0.4–0.5 kg/m^2^ per decade from 1980 until 2008, with the greatest BMI in the United States for both males and females. An estimated 9.8–13.8%% of the worldwide population were affected by obesity (BMI ≥ 30) in 2008, translating into approximately 500 million adults (older than 20 years). The obesity prevalence is greater than 20% in adults from many industrialized countries, surpassing the 30% in the United States [14,15]. Although it is unproven that obesity per se increases the risk of postoperative pulmonary complications [16,17], reports of postoperative hypoxemia, ICU admission and other resources use, and hospital length of stay, are often greater, especially in the presence of severe obesity and/or sleep apnea [18-20].

In this review, we will summarize and focus on the current-state of knowledge regarding the use of protective ventilation for intra- and peri-operative purposes for obese patients.

### Background of perioperative protective ventilation

Mechanical injury to the blood-gas barrier is the hallmark of ventilator-induced lung injury (VILI). The main determinants of VILI depend on the nature, duration, and intensity of exposure: in short, the mechanical “hit” to the lung [21,22]. Initial studies in rodent models showed that mechanical ventilation with larger tidal volumes (V_T_) causes structural pulmonary damage (volutrauma) that mimics ARDS [21-26]. This injury can begin within minutes of ventilation [22,26]. Larger V_T_ with alveolar overdistention [21-24] and cyclical opening and collapse (atelectrauma) [27] of alveoli can trigger and amplify a local inflammatory reaction of the lung (biotrauma) [28-30]. These events can potentially lead to diffuse alveolar damage characterized by pulmonary edema, recruitment and activation of inflammatory cells, local production of inflammatory mediators, and leakage of mediators into the systemic circulation [21,22,28,31-36]. Preexisting or concomitant lung alterations (i.e. underlying lung disease, systemic inflammation and/or pulmonary edema) likely make the diseased lung parenchyma much more susceptible to mechanical injury [22,26,37]. The clinical translation of these findings in animal models of VILI and ARDS has been confirmed. In patients with ARDS, a multicenter prospective ARDS Network Trial and other studies repeatedly found that, compared with conventional ventilation (with V_T_ >10 mL/kg predicted body weight, PBW), protective ventilation with lower V_T_ (6 mL/kgPBW) [1] decreases neutrophil alveolar infiltration and the levels of proinflammatory mediators in the bronchoalveolar lavage and systemically, increases the number of ventilator-free days and reduces the in-hospital mortality [1,29,30]. For this reason, in Intensive Care Unit (ICU) patients, mechanical ventilation with low V_T_ is the standard practice for preventing and managing VILI and acute respiratory distress syndrome (ARDS) [2,23].

No clear guidelines exist for setting V_T_ and optimal ventilator management in patients without ARDS. Despite controversial findings [38], several animal and human studies suggest an association between higher V_T_ and early increased inflammation and ARDS in subjects without preexisting lung disease [9,36,39-48]. Recent findings reveal improved clinical outcomes (lower incidence of ARDS, mortality) when low V_T_ ventilation is used in mechanically ventilated patients without ARDS [6,9,49]. Moreover, donor lungs from patients after brain death were better protected when receiving a low V_T_ strategy combined with alveolar recruitment maneuvers, an approach that made the lungs more likely to meet the criteria for donation [50]. Lung transplant recipients included in the protective ventilation strategy group had a 6% better survival after 6 months [50]. Thus, lung protective ventilation strategies appear to have preventative value not only in patients with healthy lungs but also in individuals receiving transplanted lungs [51].

Postoperative ARDS and other postoperative pulmonary complications, including atelectasis, pneumonia and respiratory failure, adversely impact clinical outcomes and increase hospital length of stay and medical costs [52,53]. Preventing these complications is increasingly a measure of the quality of hospital care [12]. During surgery, anesthesiologists use mechanical ventilation in patients with healthy lungs, a variety of respiratory conditions, and even in patients who are developing or will develop several other potential insults to the lung, including sepsis, trauma, lung ischemia-reperfusion, cardiac surgery, or blood transfusion [9,13,36,39-47,49]. Many of these non-ventilation insults are not preventable or avoidable, but contribute to increase the risk of postoperative ARDS development. The use of lower V_T_ ventilation is one of the few preventative measures that can be used to preserve lung health. Unfortunately, the intraoperative use of large V_T_ (greater than 10 mL/kgPBW) and no PEEP is not a rare finding, particularly in patients with obesity or short height [3-5]. The awareness and relevance of this likely unintentional practice has increased during the last few years. In particular, the recent IMPROVE trial [6], a multicenter, double-blind clinical trial, showed improved pulmonary outcomes (pneumonia, acute respiratory failure, atelectasis) and shortened hospital stays in patients ventilated for elective major abdominal surgery with a protective ventilation management approach (V_T_ 6–8 mL/kgPBW, PEEP 6–8 cmH_2_O and protocolized recruitment maneuvers) compared to a non-protective strategy (V_T_ 10–12 mL/kgPBW, PEEP 0 cmH_2_O, no recruitment maneuvers) [6]. Results from the IMPROVE study turned the focus into not only avoiding volutrauma (by using low V_T_) but also minimizing atelectrauma with adequate recruitment maneuvers and PEEP.

Another multicenter controlled study, the PROVHILO trial [8], randomized patients at risk for pulmonary complications after open abdominal surgery to receive intraoperative protective ventilation (V_T_ 8 mL/kgPBW) with either high PEEP (12 cmH_2_O and recruitment maneuvers) or low PEEP (2 cmH_2_O and no recruitment maneuvers). No difference in a composite of varied pulmonary complications (including hypoxemia or ARDS but also pneumothorax or cardiogenic pulmonary edema) during the first 5 postoperative days was observed between the groups. Intraoperatively, the PROVHILO low PEEP group required more interventions for desaturation and the high PEEP group required more interventions for hypotension. Thus, the search for optimal intraoperative ventilation settings is still incomplete. Ongoing efforts include the PROBESE study (http://www.clinicaltrials.gov/ct2/show/NCT02148692?term=probese&rank=1), a multicenter controlled trial specifically focused on the intraoperative ventilation of surgical patients with obesity.

More prospective clinical studies are needed to define the perioperative ventilation strategies for V_T_, PEEP and recruitment maneuvers that improve pulmonary outcomes, both in the general surgical and obese surgical populations.

### Perioperative pulmonary challenges related to obesity

Obese patients often present with additional pulmonary comorbidities, including airway hyper-reactivity, sleep apnea (SA), obesity hypoventilation syndrome (OHS) and pulmonary hypertension. Obesity induced airway hyper-reactivity is gaining attention as a specific type of bronchial hyper-reactivity that can be differentiated from other asthma etiologies in terms of age of onset and response to standard therapy or weight loss [54-56]. Sleep apnea is distinguished by multiple interruptions of ventilation during the sleep and their consequences (intermittent nocturnal hypoxemia and daytime tiredness). In obese patients, sleep apnea is usually from upper airway obstruction as a result of excessive soft pharyngeal tissue, rather than from a central deregulation of the respiratory drive center [57]. The fact that the time-consuming and expensive gold-standard diagnostic polysomnography is not done routinely probably contributes to a high incidence of undiagnosed sleep apnea in surgical patients [58-60]. Sleep apnea increases the risk of postoperative hypoxemia, other respiratory complications and ICU admission [18,19], and especially if untreated, also may contribute to hypertension and other cardiovascular risks [61,62]. Obesity hypoventilation syndrome (OHS) is the combination of daytime hypercapnia and sleep-disordered breathing in an obese patient, and is notably a condition that is not related to any other pulmonary or neuromuscular pathology [63,64]. OHS is frequently undiagnosed (and untreated) [65] until an acute-on-chronic respiratory failure occurs, frequently during the perioperative period [64,66]. Pulmonary hypertension often arises from the chronicity of SA or OHS, and may lead to right ventricular failure.

Aside from the previously mentioned comorbidities that can complicate the perioperative oxygenation and ventilation of surgical patients, obesity itself poses specific challenges to intraoperative airway management. Obesity and sleep apnea are frequently mentioned risk factors for difficult mask ventilation and/or tracheal intubation [67,68]. In addition to the technical challenges, the reduced functional residual capacity (FRC), increased ventilation-perfusion mismatch and respiratory comorbidities make anesthetic induction and airway management a high-risk period for hypoxemic events and other respiratory complications.

The implications of obesity on pulmonary physiology are well known [69] (Table 1). Obesity increases chest wall elastic resistance and decreases the respiratory system compliance [70,71]. Reduced respiratory system compliance is partially related to the extra adipose tissue in the chest wall but also the increased pulmonary blood volume. Most lung capacities are decreased, primarily the FRC and the expiratory reserve volume (ERV) [72-74]. The lower FRC, especially in the supine position, often leads to lung volumes lower than the closing capacity, causing ventilation-perfusion mismatch and hypoxemia. Ventilation then takes place in the less compliant portion of the pressure-volume curve, increasing the effort needed to overcome this decreased respiratory elasticity. The auto-PEEP secondary to airway closure during expiration contributes to the increased work of breathing (WOB) due to the additional ventilatory effort required by the diaphragm and other inspiratory muscles during the next inspiration [75,76]. Other factors potentially involved in the increased WOB apart from the altered respiratory mechanics are an upper airway mechanical obstruction, neuromuscular weakness, impaired gas exchange, and dampened ventilatory drive [77]. To reduce WOB, obese subjects usually adopt a breathing pattern with reduced tidal volumes and higher respiratory rates [78]. Additionally, due to the metabolism of the excess adipose tissue, obese patients have increased oxygen consumption and carbon dioxide production [70,71]. All these changes are more prominent when patients are in the supine position because increased intraabdominal pressure restricts diaphragmatic movement and lung expansion.

**Table 1:** **Changes of respiratory mechanics and functions in obese patients**

Physiological changes	Challenges for respiratory management
Excessive oro-pharyngeal adiposity	Upper airway obstruction
Increased risk of pharyngeal collapse during sleep	Frequent sleep apnea/obesity hypoventilation syndrome
Decreased compliance (chest wall > lung)	Decreased compliance during mechanical ventilation
Increased airway resistance	
Increased work of breathing	
Increase in resting VO_2_	Frequent hypoxemic events
Decrease in FRC and EELV	Atelectasis
FRC < closing capacity	Rapid oxygen desaturation
Small airway closure	
Alveolar collapse	
Ventilation–perfusion (V/Q) mismatch	
Increased PA-aO2, Decreased PaO2	

(EELV = End-expiratory lung volume; FRC = Functional residual capacity; PaO_2_ = Arterial partial pressure of oxygen; PA-aO2 = Alveolar to arterial partial pressure of oxygen; VO_2_ = Oxygen consumption; V/Q = Ventilation/perfusion).

Postoperatively, the major respiratory concerns of obese patients are related to their increased risk of hypoxemia and respiratory failure related to opioid-enhanced central respiratory depression, upper airway obstruction, and hypoventilation atelectasis. Particular focus must be directed to implementing an adequate opioid-sparing analgesia plan, encouraging deep breathing techniques, providing noninvasive positive pressure ventilation to minimize atelectasis and assuring adequate ventilation. Recent reviews offer further details on the postoperative care of surgical patients [64,68,79].

### Practices and recommendations for perioperative mechanical ventilation of the surgical patient with obesity

Several studies have been conducted about determining the best ventilatory strategies for obese patients under general anesthesia (Table 2). Pressure-controlled ventilation (PCV) is often the preferred ventilatory mode in obese patients, because of the more homogeneous distribution of delivered gas mixture and the increased possibility of avoiding alveolar distension and improving ventilation-perfusion mismatch when compared with volume-controlled ventilation (VCV). Some studies [80,81] demonstrate improved oxygenation with intraoperative PCV, compared to VCV in obese patients. However, no ventilatory mode significantly improves optimum delivered V_T_ or mean airway pressures [82-84]. There is also no information suggesting superior clinical outcomes with intraoperative PCV or VCV use in obese patients [82] and they should be, therefore, selected under adequate understanding of their different operation and characteristics to achieve the goals of lung protective ventilation and avoid both volu/barotrauma and hypoventilation. Another ventilatory mode, Pressure-Controlled Ventilation Volume-Guaranteed (PCV-VG) has been added to modern anesthesia machines within the last years. PCV-VG is a time-cycled, pressure-regulated mode with a variable inspiratory flow to achieve a preset V_T_. PCV-VG poses some theoretical advantages in the obese patient by assuring a minimum V_T_ with lower peak inspiratory pressures (PIP). However, the literature regarding its use in obese individuals is presently scarce. A very small crossover study [85] in 20 severely obese (BMI ≥ 40 kg/m^2^) adolescents or young adults receiving 20 minutes of ventilation with VCV, PVC or PCV-VG during laparoscopic bariatric surgery observed a lower PIP with PCV and PCV-VG modes, compared to VCV, but no differences in oxygenation or ventilation. Future studies are needed to evaluate the use of PCV-VG for ventilating the surgical patient with obesity.

**Table 2:** **Clinical trials comparing PCV with VCV in obese patients**

Year	Author	Intervention	n	Weight (kg)	BMI	V_T_(mL)	Height (m)	V_T_/PBW	RR (breath/min)	PIP (cmH_2_O)	Ppl (cmH_2_O)	PEEP (cmH_2_O)	Outcome	
													Oxygenation	Ventilation
2008	Cadi [80]	PCV	18	121 (21)	44 (5)	613 (91)		11 (1.4)	18 (0.5)	26 (4)	26 (4)	5	↑	↑
		VCV	18	119 (17)	45 (5)	573 (81)		10.2 (1.2)	18 (1.0)	33 (4)	27 (3)	5		
2008	De Baerdemaeker [84]	PCV	12	111.7 (19.2)	38.6 (3.6)	612 (170)	1.70 (0.12)	NA	11.8 (1.8)	25.8 (1.6)	25.8 (1.6)	5	**→**	↓
		VCV	12	117.4 (22.3)	41.4 (4.5)	645 (138)	1.68 (0.10)	NA	11.7 (1.2)	28.9 (4.2)	25.1 (3.7)	5		
2008	Hans [83]	PCV	crossover	NA	crossover	650 (104)		10.0 (1.9)	12.2 (0.5)	21.5 (4.8)	21.5 (4.8)	0	**→**	**→**
		VCV	40	NA	41.7 (5.8)	643 (100)		9.9 (1.8)	12.2 (0.5)	26.8 (5.2)	20.9 (4.6)	0		

(BMI = Body Mass Index; PBW = Predicted Body Weight; PIP = Peak Inspiratory Pressure; Ppl = plateau airway pressure; RR = Respiratory rate; V_T_ = Tidal volume; ↑ = increased; ↓ = decreased; **→** = unchanged).

Tidal volume settings require special attention in obese patients. Obese patients are more often exposed to greater V_T_ [3,5,13,86], an observation that likely reflects the practice of basing V_T_ computations on actual instead of predicted body weight. It is important to highlight, particularly in obese patients, that the desired V_T_ should be calculated based on the predicted body weight and not on the actual body weight because the increased thoracic appearance is due to excessive adipose tissue but not a greater intrathoracic (lung) volume. Despite the not-rare findings of large V_T_ used in obese patients, the clinical implications are unclear. A secondary analysis of the ARDS Network trial by O’Brien *et al.* [86] revealed that 58.6% of the studied population was overweight or obese. These obese patients presented greater initial (before study protocol) V_T_ and peak and plateau airway pressures. However, the outcomes associated with ARDS were not significantly different between obese and normal-weight patients [86]. Therefore, a greater awareness for appropriate selection of low V_T_ in obese patients is highly recommended, but further investigations are needed to determine the ideal V_T_ (and other ventilatory) settings for obese patients.

Achieving adequate ventilation with airway plateau pressures ≤30 cmH_2_O [1] is often challenging in obese surgical patients due to the decreased respiratory system compliance along with surgical-related factors (i.e. pneumoperitoneum, surgical retractors or Trendelenburg position) that further compromise lung expansion. Lewandowski *et al.* [87] suggested that greater inflation pressures may be tolerated by obese patients, possibly because the extra intrathoracic adiposity may limit lung overdistention [21]. Esophageal pressures are increased in spontaneously breathing obese individuals compared to lean subjects [74,88], which probably translates into reduced transpulmonary pressures. Ventilation guided by esophageal pressure has beneficial effects in oxygenation and compliance optimization in patients with ARDS [89], but this ventilation approach has not been attempted in obese surgical patients.

It is however well accepted that obese subjects are prone to develop atelectasis primarily in dependent lung areas, making recruitment maneuvers and application of PEEP a vital strategy to improve oxygenation and lung mechanics [90,91] (Table 3). Many studies indicate that a recruitment maneuver (RM) and PEEP reduced atelectasis and improved oxygenation in obese patients during surgery. A recent meta-analysis by Aldenkortt *et al.* [82] concluded that adding recruitment maneuvers to PEEP in these obese patients improved oxygenation and lung compliance without increasing the risk of hypotension from decreased preload. This open lung concept also seems to be potentially important in preventing the development of ventilator-induced lung injury by stabilizing alveoli and keeping them open, especially for patients undergoing major surgery. Additionally, the application of PEEP may also efficiently offset airflow limitation in the supine position and eliminate auto-PEEP without raising plateau pressure [92]. Indeed, increase in lung inflation may improve lung ventilation not only in large but also in small length scale regions even in normal lungs [93]. This is consistent with the reduction of heterogeneous mechanical forces at the microscopic level, a potential cause of VILI [94]. A recent meta-analysis [95] suggested that an open lung approach with PEEP in surgical patients improves postoperative oxygenation and decreases postoperative atelectasis without any adverse events, although this needs further confirmation. In obese patients, the efficiency of recruitment maneuvers and PEEP on postoperative outcomes such as oxygenation and pulmonary function remains controversial. The study by Talab *et al.* [91] found that obese patients receiving recruitment maneuvers followed by PEEP of 10 cmH_2_O had reduced lung atelectasis, improved intra- and post-operative oxygenation, shortened post-anesthesia care unit stay and fewer pulmonary complications than patients ventilated with lower PEEP levels. In contrast, Whalen *et al.* [96] observed that, although recruitment maneuvers followed by PEEP 12 cmH_2_O effectively increased intraoperative oxygenation, this effect disappeared 30 min after tracheal extubation. Thus, the postoperative effect and impact on clinical outcomes of these intraoperative lung recruitment efforts needs to be further studied. Ongoing studies, such as the multicenter PROBESE study mentioned earlier, may offer some answers to this question.

**Table 3:** **Clinical trials to assess the efficacy of open lung strategy**

Year	Author	Intervention	Recruitment maneuver		n	Weight (kg)	BMI	V_T_/BW	RR (breath/min)	PEEP	Outcome variables			
			Continuous pressure					(mL/kg)		(cmH2O)	Oxygenation	Atelectasis	PACU or hospital stay	Postoperative pulmonary complications
			Paw (cmH2O)	Time (s)										
2007	Chalhoub [128]	PEEP + RM40	40	15	26	130 (18)	44.4 (3.7)	10	12 (4)	8	↑	NA	NA	NA
		PEEP			26	131 (23)	45.5 (5.3)	10	13 (3)	8				
2009	de Souza [129]	PEEP + RM30	30	120	16	123.7 (20.6)	46.3 (5.0)	8–10	12–14/min	5	↗	NA	NA	NA
		PEEP + RM10/15/20	Stepwise method *1		17	136.4 (26.6)	50.5 (7.2)	8–10	12–14/min	5		NA	NA	NA
		PEEP			14	129.9 (22.4)	49.2 (6.3)	8–10	12–14/min	5		↗		
												↑		
2011	Futier [130]	PEEP + NPPV + RM	40	40	22	128 (17)	45 (5)	8	20 (20–21)	10	↗	(EELV)	NA	NA
		PEEP + NPPV			22	128 (20)	46 (2)	8	20 (20–20)	10		(EELV)	NA	NA
		PEEP			22	130 (28)	46 (4)	8	20 (18–20)	10				
2009	Reinius [131]	RM + PEEP	55	10	10	126 (9)	45 (5)	10	12/min	10	↑	↓	NA	NA
		RM + ZEEP	55	10	10	130 (13)	45 (4)	10	12/min	0	**→**	**→**	NA	NA
		PEEP			10	120 (14)	44 (3)	10	12/min	10				
2009	Talab [91]	RM + PEEP10	40	7–8	20	NA	44.5 (7.0)	8–10	NA	10	↑	↓	↓	↓
		RM + PEEP5	40	7–8	19	NA	38.3 (6.9)	8–10	NA	5	**→**	**→**	**→**	**→**
		RM + ZEEP			19	NA	41.8 (7.9)	8–10	NA	0				
2006	Whalen [96]	RM + PEEP12	Stepwise method *2		10	NA	48 (6)	12.7 (1.5)	17 (4)	12	**↑**	NA	**→**	**→**
		PEEP4			10	NA	53 (11)	11.8 (2.6)	17 (4)	4				
								LBW: lean body weight						

*1: Increase in PEEP from 5 to 10–15–20 cmH2O for 2 min each.

*2: Increasing in PEEP from 4 to 10 (over 3 breaths), 15 (3 breaths), and 20 (10 breaths).

During the early postoperative period and spontaneous breathing, obese surgical patients experience more severe alveolar collapse and impairment of gas exchange than normal-weight patients. Therefore, a head-up or sitting position, encouragement of deep breathing exercises, mobilization and incentive spirometry and continuous or bilevel positive airway pressure (CPAP/BiPAP) may prevent atelectasis and hypoxemia and reduce postoperative complications in obese patients.

Lastly, some controversy exists regarding the ideal inspired oxygen concentration. Obese patients often receive high oxygen concentrations because of the increased risk of the aforementioned perioperative hypoxemic events. Oxygen is obviously needed for adequate oxygenation and may have beneficial effects for postoperative nausea and surgical site infection [97]. For unknown reasons, administering high oxygen concentrations is associated with worse outcomes after myocardial infarction, cardiac arrest, stroke and in critically ill adults. Because high oxygen concentrations may enhance absorption atelectasis and worsen postoperative outcomes, some authors recommend maintaining inspired oxygen concentrations lower than 0.8 in obese patients [68,98]. However, in a recent meta-analysis Hovaguimian *et al.* [97] failed to find solid evidence to support this recommendation. Therefore, judicious use of oxygen to assure adequate oxygenation in obese surgical patients is prudent until more scientific knowledge is available.

**Figure 1:**
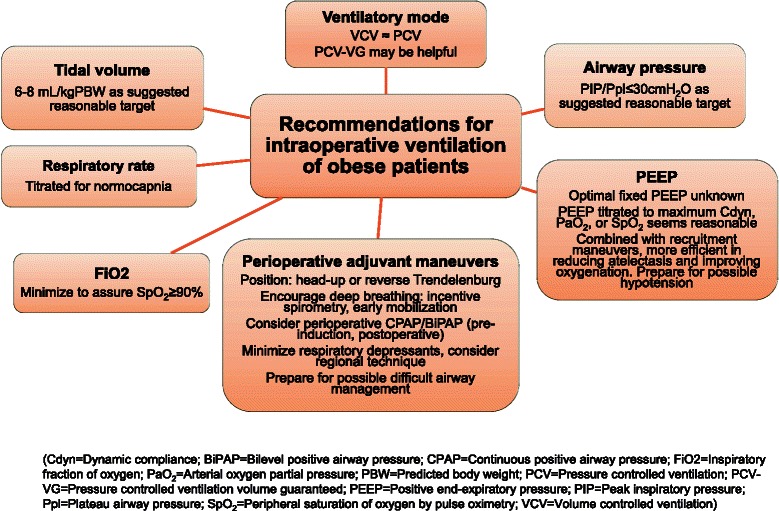
Practical recommendations for intraoperative ventilation of obese patients.

A summary of the previously described practical recommendations is included in Figure 1.

### One lung ventilation (OLV) in obese patients

Literature on the effect of OLV for the obese patient is scarce. However, the current knowledge is included herein because of the increasing number of obese patients requiring thoracic surgery.

Lung isolation for OLV is often achieved for thoracic surgery with either a double lumen tracheal tube or a single lumen tracheal tube followed by a bronchial blocker. In a recent study of obese thoracic surgery patients, Campos *et al.* found both techniques produced similar results in terms of the incidence of failed first attempts, malposition after achieving lateral decubitus position, time to lung deflation or surgical exposure [99]. The fact that only providers experienced with both techniques performed this study should be considered.

Although clinical trials testing the best ventilator settings of obese patients during thoracic surgery are limited, the essential principles of ventilator management in obese patients seem not to be different from the choices used in non-obese patients. In the past, V_T_ of 10–12 mL/kgPBW during one-lung ventilation (OLV) was recommended to maintain gas exchange and normalize arterial oxygen and carbon dioxide values. This concept is based on the previous study by Katz [100], which showed that large V_T_ produced the highest arterial oxygen tension during OLV. In fact, perioperative hypoxemia during OLV is not uncommon, which results from an intrapulmonary shunt related to collapse of the non-dependent lung and increased atelectatic areas in the dependent lung. Therefore, the primary aim during OLV is to provide adequate oxygenation and CO_2_ elimination, while the potential harmful effects of ventilatory strategy were initially disregarded. On the other hand, post-pneumonectomy pulmonary edema (PPPE) and ARDS are the most serious pulmonary complications after lung resection. In a retrospective review, Jeon *et al.* [101] observed a 12% incidence of post-pneumonectomy ARDS within the first postoperative week in series that evaluated patients with primary lung cancer. The use of large V_T_ and high airway pressures during OLV were associated with an increased risk of post-pneumonectomy ARDS. There is a growing body of evidence that the use of low V_T_ provides better outcomes after thoracic surgery. Several studies, not specific to obese surgical patients, currently recommend the use of a lung protective strategy with V_T_ of 4–6 mL/kgPBW during OLV [102-104]. Additionally, the use of a low tidal volume during OLV may be associated with less lung water content [105].

Several studies [106-108] indicate that alveolar recruitment strategies during OLV are associated with better oxygenation and decreases in dead-space variables in non-obese patients. On the other hand, excessive airway pressures in the ventilated lung during OLV can also increase pulmonary vascular resistance and shift blood flow to the non-dependent lung. Michelet *et al.* [109] found that administering 5 and 10 cmH_2_O PEEP was associated with improved oxygenation and continuous lung volume recruitment while giving 15 cmH_2_O PEEP caused overdistention and increased shunt compared with the other PEEP levels. Thus, although the use of PEEP is crucial to treat and prevent atelectasis and hypoxemia, it must be used with careful attention to the possibility of causing lung overdistention, possibly producing or contributing to ventilator-induced lung injury. In particular, the probability of coexistent auto-PEEP in patients with severe chronic obstructive pulmonary disease (COPD) presenting for lung resection needs to be considered. The best level of PEEP during OLV remains unclear but there is wide variation in individual pulmonary responses to the application of PEEP [110,111]. In a recent study by Ferrando *et al.* [112], applying individualized PEEP in a PEEP Decrement Trial resulted in better oxygenation and lung mechanics after an alveolar recruitment maneuver than administering a standardized 5 cmH_2_O of PEEP. Obese patients tend to suffer from alveolar collapse due to their decreased chest wall compliance. Therefore, in obese patients, the setting of optimal PEEP level to keep the lung open may be crucial especially because an inadequate PEEP level cannot prevent alveolar re-collapse after an alveolar recruitment maneuver, and the latter would be also expected to occur in thoracic surgery. When performing recruitment maneuvers, attention should be paid to their potential adverse effects including temporary desaturation, decreased preload, hypotension, arrhythmias, and barotrauma.

### Postoperative ventilation in obese patients in the ICU setting

Most likely, as a consequence of the previously mentioned effects of obesity on lung physiology, frequently associated respiratory comorbidities and increased risk of atelectasis, obese surgical patients have a greater risk of respiratory failure and other postoperative pulmonary complications [73,113]. Several general care recommendations in the postoperative care of obese surgical patients have been proposed to decrease the risk of atelectasis [68]. Head-up sitting position, encouragement of deep breathing and the use of continuous positive airway pressure (CPAP) may improve postoperative lung mechanics and reduce postoperative complications in patients undergoing surgery [68,114]. Postoperative admission of obese surgical patients to the ICU or intermediate care units is not unusual for a more intense ventilatory monitoring.

When postoperative ventilation is needed in obese surgical patients, ventilatory practice in the ICU tends to follow recommended protective ventilation strategies, albeit not completely [13,115] but more tightly than in the operating room [3,116]. However, Gajic *et al.* [49] reported that 24% of ICU patients with normal lungs ventilated for 2 days or longer develop ARDS [49]. In this study, the main risk factors for ARDS were large tidal volumes (OR 1.3 for each mL/kg above 6 mL/kgPBW), blood transfusion, and restrictive lung disease [49]. Nonetheless, the impact that obesity poses on the risk of ARDS is still controversial. Although some authors [86,117,118] observed a similar or increased incidence of ARDS in severely obese patients, the clinical outcomes (i.e. mortality, hospital length of stay) of obese patients were similar to those in non-obese patients. Furthermore, other studies [119-121] found a decreased incidence and/or mortality from ARDS in obese patients, and decreased plasma concentrations of inflammatory mediators (IL-6, IL-8) during ARDS [122] in obese patients compared to normal-weight patients. It seems clear that obese patients, once they present with respiratory failure in the ICU, usually require longer durations of mechanical ventilation [123,124]. Therefore, the effect of different degrees of obesity on incidence and outcomes of ARDS and a wide array of cardiovascular and metabolic comorbidities is still not well explained. Overweight and mildly obese and otherwise-healthy patients may actually not be at increased risk of ARDS as initially expected [86,119-122,125], phenomenon that has been termed the Obesity ARDS Paradox. [126,127]. Further confirmation of this intriguing paradox and its underlying mechanisms are needed, but it might hold new insights into the pathophysiology, diagnosis, treatment and prevention of ARDS.

## Conclusions

The increasing number of obese patients requiring surgery demands a better understanding of the particular challenges that obesity poses on mechanical ventilation. Obese patients present specific lung physiology and mechanics characteristics, frequent respiratory comorbidities and increased risk of postoperative pulmonary complications. Intraoperatively, lung protective ventilation with low tidal volumes, recruitment maneuvers with greater PEEP levels and the judicious use of oxygen concentrations are recommended. Focused postoperative care seeking to minimize atelectasis formation is critical. Further research is needed to identify the ideal perioperative respiratory care needed to enhance the outcomes and minimize postoperative pulmonary complications of obese surgical patients.

## Abbreviations

ARDS: Acute respiratory distress syndrome
BiPAP: Bilevel positive airway pressure
BMI: Body mass index
Cdyn: Dynamic compliance
CPAP: continuous positive airway pressure
EELV: End-expiratory lung volume
ERV: Expiratory reserve volume
FiO_2_: Inspiratory fraction of oxygen
FRC: Functional residual capacity
ICU: Intensive care unit
OHS: Obesity hypoventilation syndrome
OLV: One lung ventilation
PaO2: Arterial partial pressure of oxygen
PA-aO2: Alveolar to arterial partial pressure of oxygen
Paw: Airway pressure
PBW: Predicted body weight
PCV: Pressure-controlled ventilation
PCV-VG: Pressure-controlled ventilation volume-guaranteed
PEEP: Positive end-expiratory pressure
PIP: Peak inspiratory pressures
Ppl: Plateau airway pressure
RM: Recruitment maneuver
SA: Sleep apnea
SpO_2_: Peripheral saturation of oxygen by pulse oximetry
VCV: Volume-controlled ventilation
VILI: Ventilator-induced lung injury
VO_2_: Oxygen consumption
V/Q: Ventilation/perfusion
V_T_: Tidal volume
WOB: Work of breathing
